# Supplementary material for: The interactome of intact mitochondria by cross-linking mass spectrometry provides evidence for coexisting respiratory supercomplexes
Source: Mol Cell Proteomics. 2017 Dec 8;17(2):216–32. doi: 10.1074/mcp.RA117.000470 (PMC5795388; doi:10.1074/mcp.RA117.000470)
Supplement: Supplemental Data [file supp_RA117.000470_133922_0_supp_23978_fzffwf.zip › spectra_annotation/mito_DR_spectra_annotation/17-1-6-1-30-1.pdf]

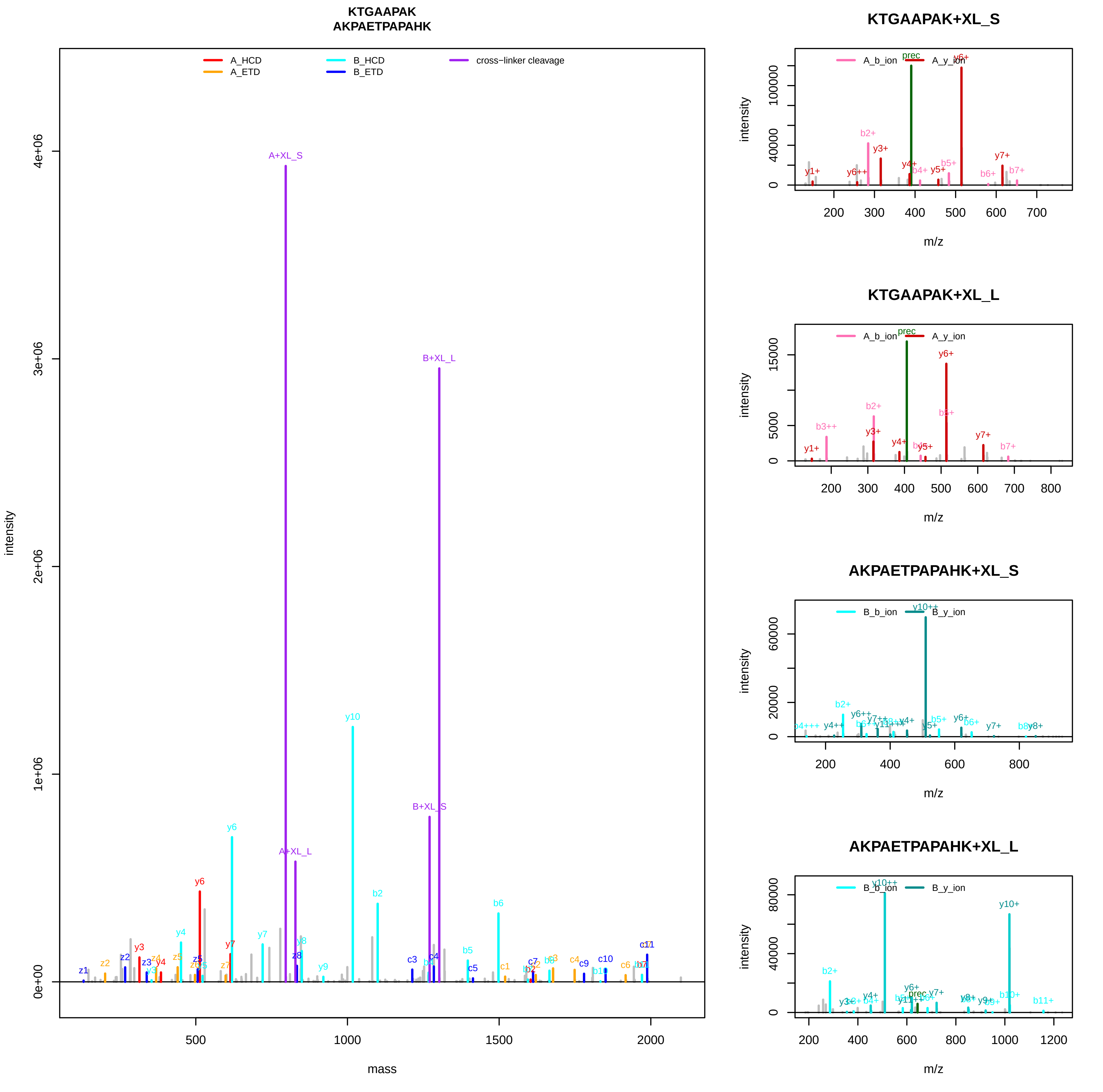

KTGAAPAK
AKPAETPAPAHK
A_HCD
A_ETD
B_HCD
B_ETD
cross−linker cleavage
A+XL_S
B+XL_L
B+XL_S
A+XL_L
y10
y6
b2
b6
y4
y7
y8
y9
b5
c3
c4
c5
c7
c9
c10
c11
c1
c6
z1
z2
z3
z5
z8
y3
y7
intensity
mass
0e+00
1e+06
2e+06
3e+06
4e+06
500
1000
1500
2000
KTGAAPAK+XL_S
KTGAAPAK+XL_L
AKPAETPAPAHK+XL_S
AKPAETPAPAHK+XL_L
A_b_ion
A_y_ion
B_b_ion
B_y_ion
prec
y10++
y10+
m/z
intensity
